# BRET-based biosensors for SARS-CoV-2 oligonucleotide detection

**DOI:** 10.3389/fbioe.2024.1353479

**Published:** 2024-06-03

**Authors:** Asfia Sultana, Anupriya M. Geethakumari, Zeyaul Islam, Prasanna R. Kolatkar, Kabir H. Biswas

**Affiliations:** ^1^ Division of Biological and Biomedical Sciences, College of Health and Life Sciences, Hamad Bin Khalifa University, Education City, Qatar Foundation, Doha, Qatar; ^2^ Diabetes Center, Qatar Biomedical Research Institute, Hamad Bin Khalifa University, Education City, Qatar Foundation, Doha, Qatar

**Keywords:** biosensor, bioluminescence, bioluminescence resonance energy transfer, COVID-19, SARS-CoV-2, molecular beacon

## Abstract

The need for the early detection of emerging pathogenic viruses and their newer variants has driven the urgent demand for developing point-of-care diagnostic tools. Although nucleic acid-based methods such as reverse transcription-quantitative polymerase chain reaction (RT-qPCR) and loop-mediated isothermal amplification (LAMP) have been developed, a more facile and robust platform is still required. To address this need, as a proof-of-principle study, we engineered a prototype—the versatile, sensitive, rapid, and cost-effective bioluminescence resonance energy transfer (BRET)-based biosensor for oligonucleotide detection (BioOD). Specifically, we designed BioODs against the SARS-CoV-2 parental (Wuhan strain) and B.1.617.2 Delta variant through the conjugation of specific, fluorescently modified molecular beacons (sensor module) through a complementary oligonucleotide handle DNA functionalized with the NanoLuc (NLuc) luciferase protein such that the dissolution of the molecular beacon loop upon the binding of the viral oligonucleotide will result in a decrease in BRET efficiency and, thus, a change in the bioluminescence spectra. Following the assembly of the BioODs, we determined their kinetics response, affinity for variant-specific oligonucleotides, and specificity, and found them to be rapid and highly specific. Furthermore, the decrease in BRET efficiency of the BioODs in the presence of viral oligonucleotides can be detected as a change in color in cell phone camera images. We envisage that the BioODs developed here will find application in detecting viral infections with variant specificity in a point-of-care-testing format, thus aiding in large-scale viral infection surveillance.

## Introduction

Viruses are a major threat to human health, accounting for ∼30% of infections worldwide, including respiratory diseases, causing serious socioeconomic challenges every year (Iversen, 2018). Among these, coronaviruses are of significantly greater concern due to their recurrent emergence as highly virulent pathogenic strains from a relatively benign human pathogen (Wu et al., 2020). These include the members of the beta coronavirus family, such as the Middle East respiratory syndrome coronavirus (MERS-CoV) and severe acute respiratory syndrome coronavirus (SARS-CoV) and SARS-CoV-2, the causative agent of COVID-19 (coronavirus disease 2019) (Feng et al., 2020; Younes et al., 2020; Zhu et al., 2020). The later has already caused more than 6 million deaths and more than 700 million infections, as reported up to March 2024. Importantly, several novel variants of SARS-CoV-2 have emerged with increased infection potential and resistance to antibody-mediated neutralization (Tegally et al., 2021; Torretta et al., 2021; Voloch et al., 2021; Philip et al., 2023) (https://covid19.who.int/).

One of the key strategies to deal with viral infections, in addition to the development of effective vaccines such as those targeting the viral structural proteins and therapeutic approaches such as pharmacologically inhibiting proteins that play a critical role in viral replication, is the early detection of the virus (Habli et al., 2020; Qin et al., 2020). This facilitates the containment of its spread rather than directly impacting patient survival rates, thereby preventing further disease transmission and mitigating healthcare expenses. In this regard, technologies that allow the robust detection of viruses in patient or environmental samples are critical (Feng et al., 2020). Antibody-based tests, also known as serological tests, detect the presence of the antibodies produced by the immune system in response to infections (viral) (Fox et al., 2022). Antigen tests detect the presence of specific proteins that are a part of the virus. Antigen tests are often used for diagnosing active infections as they can detect the presence of viruses (Grandien, 1996). Nucleic acid-based tests, also known as molecular tests, detect the presence of specific genetic material, such as DNA or RNA, from a pathogen such as a virus or bacterium (Guglielmi, 2021; He et al., 2022; Ju et al., 2022). Although the former two tests can be implemented in a point-of-care testing (POCT) format, they often do not possess the sensitivity necessary for identifying infections with low viral titers. Moreover, there are instances where the antibodies may not detect antigens, such as the SARS-CoV-2 spike, which has been reported to mutate frequently in variants such as Delta, Beta, and Omicron (Li et al., 2020; Ahmed et al., 2022).

On the other hand, nucleic acid-based methods such as real-time reverse transcription-quantitative polymerase chain reaction (RT-qPCR), which is considered the industry gold standard, provide significantly higher sensitivities and specificities (Varlamov et al., 2020; Torretta et al., 2021; Fang et al., 2022). However, they require specialized laboratory facilities and costly equipment, making them less suitable for rapid large-scale deployment in some contexts (Younes et al., 2020). To circumvent these issues, several variations of nucleic acid detection techniques have been developed. For instance, loop-mediated isothermal amplification (LAMP) (Dao Thi et al., 2020; Wang et al., 2021) is relatively faster but can lead to false positive results due to non-specific amplification and primer interactions. On the other hand, CRISPR-based methods such as those using the RNA-cleaving Cas13 nuclease (Joung et al., 2020) require relatively expensive, fluorescently labeled RNA-based reporters and additional instruments for fluorescence measurements. Alternative strategies such as PHAsed NASBA-Translation Optical Method (PHANTOM) based on a DNA toe-hold structure coupled to *in vitro* protein translation and detection (Chakravarthy et al., 2021) require significantly expensive protein synthesis that is in-built into the assay. Therefore, there is a demand for the development of biosensing platforms that possess high specificity and sensitivity, are deployable at a large scale, and are easily adaptable to constantly evolving viral genomes (Mao et al., 2022; Van Ngoc et al., 2022; Dong et al., 2023; Nelson-Mora et al., 2023).

**FIGURE 1:**
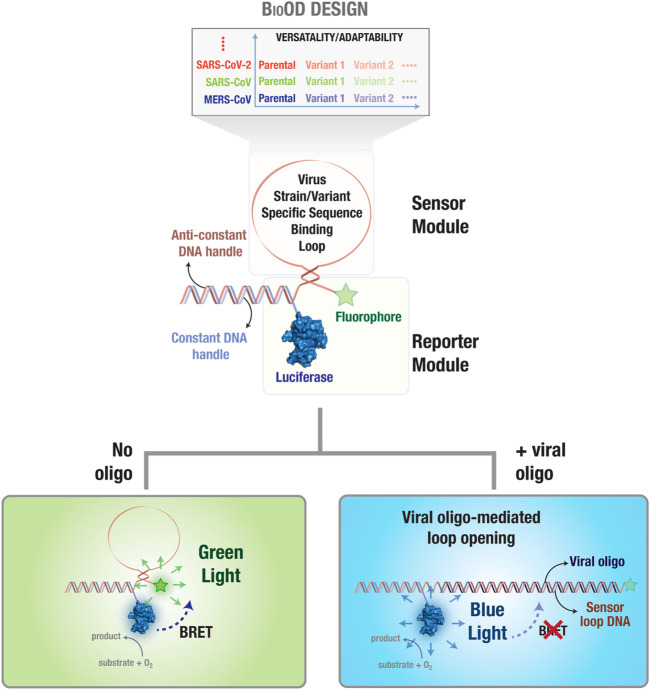
Bioluminescence resonance energy transfer (BRET)-based biosensor for viral oligonucleotide detection (BioOD). Schematic representation of the BRET-based biosensor (BioOD) design for SARS-CoV-2 parental and Delta variant nucleic acid detection. The biosensor consists of a reporter module (constant DNA handle conjugated to a luciferase protein) and a DNA stem loop-based sensor module (viral strain and variant nucleic acid, RNA in this case, binding oligonucleotide sequence). Close positioning of the luciferase (NLuc; BRET donor) and fluorophore (BRET acceptor) proteins results in significant BRET in the absence of a viral oligonucleotide. Binding of the viral oligonucleotide will result in the dissolution of the loop, leading to a decrease in BRET.

In the current study, we developed a biosensor platform, bioluminescence resonance energy transfer (BRET)-based biosensor for SARS-CoV-2 oligonucleotide detection (BioOD) (Figure 1), which combines the benefits of the high signal-to-noise ratio of BRET-based reporters and the sensitivity and specificity provided by oligonucleotide binding (Geethakumari et al., 2022a). BRET relies on the non-radiative resonance energy transfer from a luciferase donor (emits light by oxidizing its substrate) to a fluorescent acceptor with suitable spectral overlap (Biswas et al., 2008; Dale et al., 2019; Ong et al., 2020). Importantly, BRET efficiency varies inversely with the sixth power of the distance between the donor and the acceptor, and thus, a small increase in the distance between the two results in a large decrease in the BRET efficiency. We successfully used this technique to develop highly sensitive and specific biosensors (Biswas et al., 2008; Biswas and Visweswariah, 2011; Biswas and Visweswariah, 2017; Geethakumari et al., 2022b), including one for monitoring SARS-CoV-2 main protease activity (Geethakumari et al., 2022a; Moovarkumudalvan et al., 2022; Jan et al., 2023). For engineering a BRET-based oligonucleotide detection platform (biosensors) for SARS-CoV-2 parental and Delta variants, we explored the utilization of both *Gaussia* luciferase (GLuc) and NanoLuc (NLuc) luciferase as the energy donor and utilized NLuc due to its high bioluminescence that will aid in the utilization of the biosensor in a point-of-care diagnostics tool. NLuc was then conjugated with SARS-CoV-2 variant-specific molecular beacon oligonucleotides functionalized with an appropriate fluorophore. The assembled BioODs showed rapid response to complementary, specific oligonucleotides, but not with non-specific oligonucleotides, in a concentration-dependent manner. Importantly, the change in BRET efficiency in the form of a shift in the bioluminescence spectra could be detected through cell phone camera imaging.

## Materials and methods

### Target sequence of SARS-CoV-2 and Delta variant analysis

The parental (Wuhan strain) and Delta variant sequences with accession ID–NC_045512.2 and EPI_ISL_1704637 were downloaded from the NCBI virus database (https://www.ncbi.nlm.nih.gov/genome/viruses/) and GISAID (https://gisaid.org/) database, respectively. For Wuhan/SARS-CoV-2, the “GTTAATAGTTAATAGCG” nucleotide sequence was chosen as the target sequence. The Delta variant containing the D950N (G24410A) mutation in the spike protein region (S2) nucleotide sequence “GCA​CTT​GGA​AAA​CTT​CAA​A” was chosen as the target sequence for designing the BioOD (Zhan et al., 2022). These target sequences were checked for any internal binding using the oligonucleotide analyzer tools (https://www.idtdna.com/pages/tools/oligonucleotideanalyzer) (Owczarzy et al., 2008) and Oligonucleotide Calc (http://biotools.nubic.northwestern.edu/OligonucleotideCalc.html) (Kibbe, 2007) and by aligning the sequences using the alignment tool ClustalW (https://www.ebi.ac.uk/Tools/msa/clustalo/) (Thompson et al., 1994).

### Mutagenesis, expression, and purification of GLuc and NLuc luciferase

Two different plasmid constructs were designed for the expression and purification of SNAP-GLuc-His_10_ and NLuc(C166S/G182C)-His_10_ luciferase proteins. The gene coding for GLuc and NLuc luciferases was generated in pET22b expression vectors, with one vector having GLuc fused with the SNAP-tag on the N-terminal and His-tag toward the C-terminal (GenScript, Singapore). The other vector contained the NLuc, where the native cysteine was mutated to serine (C164S) and the glycine (not a part of NLuc but present after the end of NLuc) was mutated to cysteine (G182C) for site-specific DNA conjugation using multisite-directed mutagenesis according to the manufacturer’s protocol (GenScript, Singapore). The plasmid encoding for GLuc and the mutated NLuc was subsequently transformed in *E. coli* BL21 and cultured in LB medium supplemented with 30 mg/L ampicillin in a total volume of 500 mL. At OD_600_ = 0.6, protein expression was induced by the addition of 100 μM isopropyl β-D-1-thiogalactopyranoside (IPTG) overnight at 18°C (Biswas et al., 2015a; Biswas et al., 2015b; Biswas et al., 2016; Biswas and Groves, 2016; Biswas et al., 2018; Geethakumari et al., 2022a; Geethakumari et al., 2022b; Guo et al., 2022). Subsequently, the cells were harvested by centrifugation for 10 min at 10,000 g and lysed by resuspending the pelleted cells in the protein extraction reagent. The soluble fraction was obtained by centrifugation for 40 min at 40,000 g. Finally, SNAP-GLuc-His_10_ and NLuc(C166S/G182C)-His_10_ luciferases were purified from the soluble fraction by Ni^2+^-affinity chromatography, and the buffer was changed to the storage buffer [50 mM Tris-HCl (pH 7.4), 150 mM NaCl, 10% glycerol (v/v), 1 mM dithiothreitol [DTT], 0.1 mM phenylmethylsulfonyl fluoride (PMSF), and 0.02% sodium azide (w/v)].

### BioOD design

BioOD is a single-stranded oligonucleotide sequence comprising the constant handle (5′ GTG​ATG​TAG​GTG​GTA​GAG​GAA 3′), anti-handle (5′ TTC​CTC​TAC​CAC​CTA​CAT​CAC 3′), stem sequences (5 bp), and a loop region containing the complementary sequences of the target SARS-CoV-2 (Wuhan/parental) or the Delta variant (B.1.617.2) (Engelen et al., 2017). The 5′ end of the handle DNA is maleimide-modified, and is conjugated with the cysteine-modified NLuc protein (NLuc- C166S/G182C)-His_10_; BRET donor) (Chen et al., 2017). Anti-handle DNA is the complementary oligonucleotide sequences to the handle DNA, which folds as a stem and a loop region having the target sequence of the parental and Delta variant, while the 3′-end is conjugated to the fluorophore, which is Alexa Fluor 488 (acceptor) in the case of the parental and Alexa Fluor 532 (acceptor) for the Delta variant synthesized (Integrated DNA Technologies [IDT]; Iowa, United States) (Supplementary Figure S2).

### Deprotecting maleimide-conjugated oligonucleotides

The maleimide-conjugated handle oligonucleotides synthesized (Integrated DNA Technologies [IDT]; Iowa, United States) were functionalized by deprotecting them using acetonitrile and toluene prior to conjugation with the luciferase protein. The deprotection of the protected maleimide oligonucleotide is a retro Diels–Alder reaction, which generates the reactive maleimide (Gil Alvaradejo et al., 2018). Briefly, the oligonucleotides (aqueous solution) were first lyophilized and again dried by co-evaporation with anhydrous acetonitrile and later co-evaporated thrice with anhydrous toluene. After drying, the oligonucleotides were suspended in 2 mL anhydrous toluene and kept at 90°C for 4 h for complete evaporation (Gobbo and Workentin, 2012), as per the manufacturer’s protocol (Mattioli et al., 2015; Paris et al., 2015). Maintaining the anhydrous condition is the key during the deprotection method because the presence or addition of water or any significant moisture may lead to incomplete deprotection, hydrolysis. After the complete evaporation of toluene, the deprotected oligonucleotides tend to be very unstable, thus demanding the conjugation of the protein at the earliest (Marchán et al., 2006).

### Maleimide–ODN-NLuc(C166S/G182C)-His_10_ conjugation

Prior to the protein–DNA conjugation, the cysteine-modified NLuc protein was purified by PD-10 columns (Sigma-Aldrich) to remove the reducing agent DTT. The biosensors were assembled by adding the deprotected maleimide oligonucleotides in PBS (100 mM sodium phosphate and 150 mM NaCl, pH 7.2) to a final concentration of 1 mM and adding 3-fold molar excess of purified cysteine-modified NLuc and incubating it for 2 h at room temperature for enhanced conjugation.

### Maleimide–ODN-NLuc purification

The ODN-NLuc conjugate was purified by Ni^2+^-affinity chromatography and using a PD-10 column to remove the excess handle oligonucleotide and unreacted NLuc, respectively. Briefly, for Ni^2+^-affinity chromatography, the reaction mixture was loaded on a prepacked His-binding resin column, and the excess of handle oligonucleotide was removed by washing the column with 500 μL wash buffer (20 mM Tris-HCl, 250 mM NaCl, and 60 mM imidazole, pH 7.9). A total of 3.5 mL of elution buffer (20 mM Tris-HCl, 250 mM NaCl, and 500 mM imidazole, pH 7.9) was added to remove the unreacted NLuc and ODN-NLuc. The elution fractions were pooled, and the buffer was exchanged to a low-ionic strength buffer (20 mM Tris-HCl, pH 7.0) using a PD-10 desalting column, and directly unreacted NLuc was eluted by washing the anion exchange column with a low-ionic strength buffer (20 mM Tris-HCl and 250 mM NaCl, pH 7.0). Finally, ODN-NLuc was eluted with a high-ionic strength buffer (20 mM TrisHCl and 1 M NaCl pH 7.0)

### BRET and fluorescence measurements

*In vitro* BRET-based assays were performed by incubating the SARS-CoV-2 parental and Delta variant BioOD in Tris-buffered saline (TBS) for characterizing the proper assembly of the BioOD using a Tecan SPARK^®^ multimode microplate reader. Bioluminescence spectral scans were performed from wavelengths of 380 to 664 nm with an acquisition time of 400 ms for each wavelength to determine the relative emissions from NLuc (donor) and Alexa Fluor 488 and 532 (acceptor) and quantify BRET, which is expressed as the ratio of the emissions at 516 to 467 nm for the SARS-CoV-2 parental strain and 566 to 467 nm for the Delta variant.

### *In vitro* BRET-based assay

*In vitro* BRET assays were performed by incubating the SARS-CoV-2 and Delta variant BioODs with their respective complementary oligonucleotides at different concentrations (10^−5^ to 10^−11^ M) in 1 × TBS, and BRET was monitored by acquiring the bioluminescence spectra of the respective samples. Percentage changes in BRET were determined after subtracting a background BRET ratio of 0.2, which was determined similarly using a recombinantly purified NLuc protein, and the BRET percentage change was also calculated for all the five different complementary oligonucleotides of both the SARS-CoV-2 and the Delta variant (Geethakumari et al., 2022b).

### Cell phone camera-based detection of the viral DNA oligonucleotide

Viral DNA oligonucleotides were detected by incubating the BioODs with their respective oligonucleotides complementary to both stems, and the loop of the molecular beacons used to assemble the BioODs in a 96-wellwhite plate and bioluminescence images were acquired post-incubation using a cell phone camera in a dark chamber upon the addition of the luciferase substrate.

### Data analysis and figure preparation

GraphPad Prism (GraphPad Software, La Jolla, CA, United States; www.graphpad.com), in combination with Microsoft Excel, was used for data analysis and graph preparation. Figures were assembled using Adobe Illustrator.

## Results and discussion

### BioOD design

In order to engineer SARS-CoV-2 parental and Delta variant-specific BioODs (Figure 1), we designed sensor modules consisting of molecular beacons (Kim et al., 2008; Sherrill-Mix et al., 2021), which are single-oligonucleotide sequences that fold as a stem–loop structure and can be tuned for specificity and sensitivity by altering the oligonucleotide length and sequence (Han et al., 2013). Each of the molecular beacons were functionalized with an organic fluorophore (BRET acceptor) on one side and a luciferase protein (BRET donor) through the hybridization of a constant DNA handle sequence, which was covalently conjugated to the luciferase protein through thiol–maleimide chemistry, and an anti-handle DNA sequence that appended to the molecular beacons (Figure 1) (Dhawan et al., 2022; Zhan et al., 2022). Thus, in the absence of any target oligonucleotide binding, the stem–loop structure of the molecular beacon will position the fluorophore (BRET acceptor) close to the luciferase protein (BRET donor), resulting in a highly efficient resonance energy transfer, leading to high BRET (Figure 1) (Severins et al., 2018; Moore et al., 2021). However, upon the binding of a complementary target oligonucleotide to the molecular beacon, the stem–loop structure of the molecular beacon in the BioOD will be disrupted, leading to an increase in the distance between the fluorophore and the luciferase and a consequent decrease in BRET, i.e., an increase in blue-light emission (Figure 1).

The molecular beacons used for constructing the BioOD for both the SARS-CoV-2 parental and Delta variants were designed *in silico*. A typical molecular beacon comprises a stem, a loop region, a fluorophore, and a quencher, and it folds to form a stem–loop hairpin structure (Tyagi and Kramer, 1996). The loop is a 15–30-bp region of the molecular beacon, which is complementary to the target sequence (Monroy-Contreras and Vaca, 2011). In our study, the loop region of the parental beacons (Supplementary Figure S1A) has the target sequence (GTT​AAT​AGT​TAA​TAG​CG) specific to SARS-CoV-2, while the BioOD for the Delta variant (Supplementary Figure S1B) has the loop region containing the Delta variant target sequence (GCA​CTT​GGA​AAA​CTT​CAA​A) with D950N (G24410A) mutation in the spike protein region (S2). The stem in a molecular beacon is formed of complementary sequences on both sides of the loop and are typically 5–7 nucleotides long. For an optimal stem design, it is crucial to maintain the selectivity and a fast rate of hybridization (Huang and Martí, 2012). Short stems of 5–6 bp with a free energy change in hybridization (∆G) of approximately −1.5 to −2 kcal/mol and higher GC content of 50%–60% are preferred. Longer stems, on the other hand, can slow down the response time of the molecular beacon and fluctuate the stability. We utilized a 5-bp-long stem with a nucleotide sequence of ACAGC-GCTGT and its complementary sequence for designing the molecular beacons of both the parental and Delta variants (Engelen et al., 2017).

Furthermore, the stem and loop nucleotide sequences were appended to the anti-handle DNA, which is complementary to the handle DNA covalently conjugated to the NLuc. Finally, the 3′ end of the anti-handle DNA was conjugated with Alexa Fluor 488 in the parental and Alexa Flour 532 in the Delta variant BioOD. The molecular beacon structures with these components were predicted using the Mfold web server, a tool specializing in analyzing DNA and RNA folding (Zuker, 2003). To ensure accuracy and efficiency, numerous molecular beacon structures were generated. Structures with low ∆G of −1.5 kcal/mol and high ∆G values above −4 kcal/mol and those displaying multiple secondary structures were not considered further. A ∆G value within this range indicates that the hairpin structure formed by the molecular beacon is stable enough to exist in its closed conformation (without the target), yet it is still sensitive to opening upon target binding. This sensitivity is crucial for the molecular beacon to efficiently undergo conformational changes in the presence of its target molecule, leading to a measurable signal change (Vet and Marras, 2005). Ultimately, beacons with a ∆G value of −3.07 kcal/mol were selected for both the parental and Delta variant BioODs (Supplementary Figures S1A and S1B), ensuring their stability and functionality.

### Characterizing the luciferase protein for BioOD assembly

**FIGURE 2:**
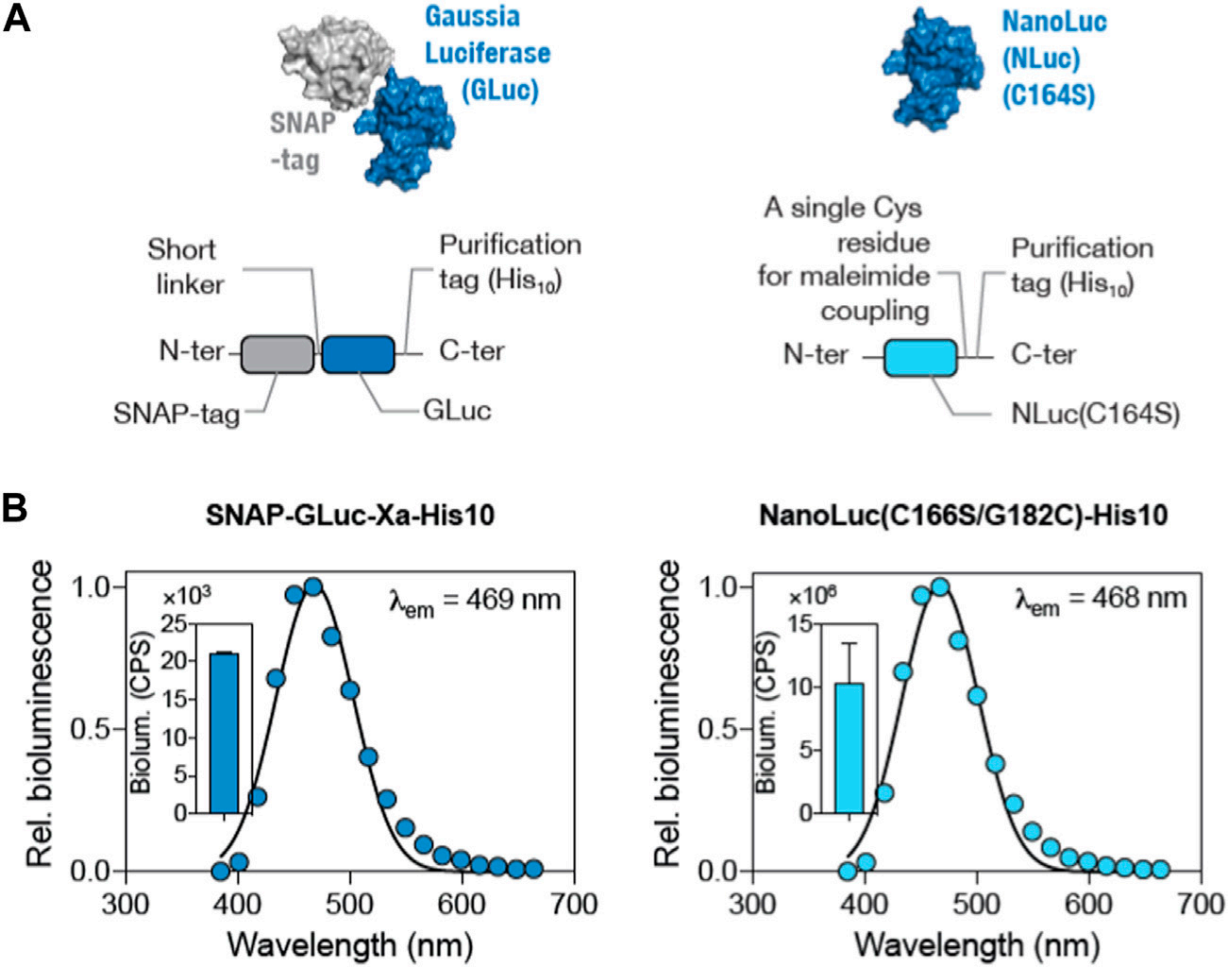
Comparison of GLuc and NLuc as the BRET donor luciferase in the BioOD. **(A and B)** Surface and schematic representation of SNAP-GLuc-His_10_ (left panel) and NLuc(C166S/G182C)-His_10_ (right panel) constructs. **(B)** Graph showing bioluminescence spectra (left panel) of the SNAP-GLuc and NLuc(C166S/G182C) (right panel) proteins. Insets show the total bioluminescence of the proteins.

In order to decide on the better luciferase protein for the protein–DNA-conjugated BioODs, two luciferase proteins, GLuc and NLuc, were characterized for their bioluminescence activity (Azad et al., 2021). Both GLuc and NLuc have been widely used in many types of BRET assays. GLuc is relatively stable and compatible with the relatively cheaper luciferase substrate, coelenterazine-h. On the other hand, NLuc has been reported to be smaller in size, possesses high luminescence, and displays high chemical and thermal stability compared to other luciferase proteins (Altamash et al., 2021). Importantly, it has been shown to be an efficient BRET donor in a number of BRET-based assays (Engelen et al., 2017; Besson et al., 2022; Geethakumari et al., 2022b). To characterize the luciferases, we generated plasmid constructs expressing SNAP-GLuc and NLuc(C166S/G182C) (Supplementary Figure S2) (coding gene sequence available in the Supplementary Material) with a C-terminal His_10_-tag and expressed and purified the luciferases using Ni^2+^-affinity chromatography, followed by gel filtration chromatography. The GLuc protein was fused with the SNAP-tag on the N-terminal and His_10_-tag on the C-terminal (Figure 2A). The NLuc protein was cysteine-modified with a C166S mutation to remove the original C residue and a G182C mutation to insert a C residue for thiol–maleimide conjugation (Figure 2B) (Engelen et al., 2017). SDS-PAGE analysis was performed to confirm the purity of both GLuc and NLuc luciferases. Bioluminescence spectra of the purified SNAP-GLuc and NLuc(C166S/G182C) proteins were measured upon adding the luciferase substrate (England et al., 2016; Geethakumari et al., 2022a). The expected emission peaks at 469 nm and 468 nm for GLuc and NLuc, respectively (Figure 2B), were observed. However, the total bioluminescence for GLuc was found to be ∼10^4^ counts per second (CPS) (Figure 2B, inset; left panel), while the total bioluminescence for NLuc was found to be ∼10^7^ CPS (Figure 2B, inset; right panel) (Sun et al., 2016; Biswas et al., 2008). Based on the higher bioluminescence, we selected NLuc as the BRET donor for assembling the BioODs.

### SARS-CoV-2 parental and Delta variant-specific BioOD assembly

**FIGURE 3:**
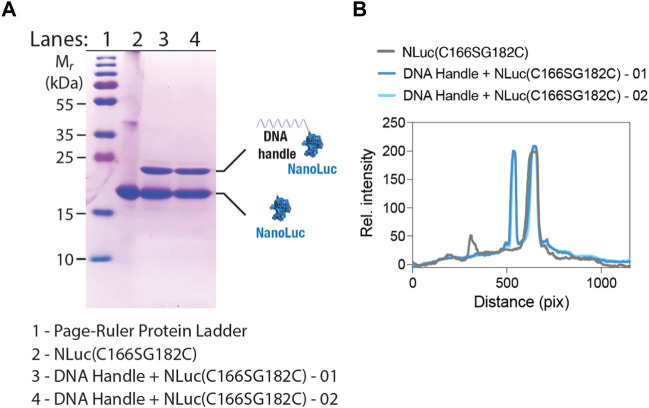
SDS-PAGE shows NLuc-constant handle DNA oligonucleotide conjugation. **(A)** SDS-PAGE image showing a shift in the electrophoretic mobility of the NLuc(C166S/G182C)-His_10_ protein after handle DNA conjugation. **(B)** Graph showing line-scan intensity profiles of the lanes in image **(A)**. Numbers 01 and 02 in panels A and B indicate reaction numbers (performed twice).

The first step in the assembly of the BioOD for the parental and Delta variant of SARS-CoV-2 required the conjugation of the maleimide-functionalized constant handle DNA oligonucleotide to the C182 residue in the NLuc(C166S/G182C)-His_10_ protein through the thiol–maleimide reaction (see Materials and Methods for details) (Kabir et al., 2018). The maleimide-functionalized constant handle DNA and NLuc(C166S/G182C) conjugation was confirmed through the electrophoretic mobility using the SDS-PAGE analysis (Guo et al., 2022), which revealed a shift in the electrophoretic mobility of the conjugated DNA handle-NLuc(C166S/G182C) compared to the non-conjugated NLuc(C166S/G182C) protein (Figure 3A). We quantified the level of conjugation using the SDS-PAGE gel image (Figure 3B) and found the peak for the conjugated DNA handle–NLuc(C166S/G182C) to be similar to that of the non-conjugated NLuc(C166S/G182C), suggesting that approximately half of the protein was conjugated. The conjugated DNA handle–NLuc(C166S/G182C) was further purified through anion exchange chromatography.

**FIGURE 4:**
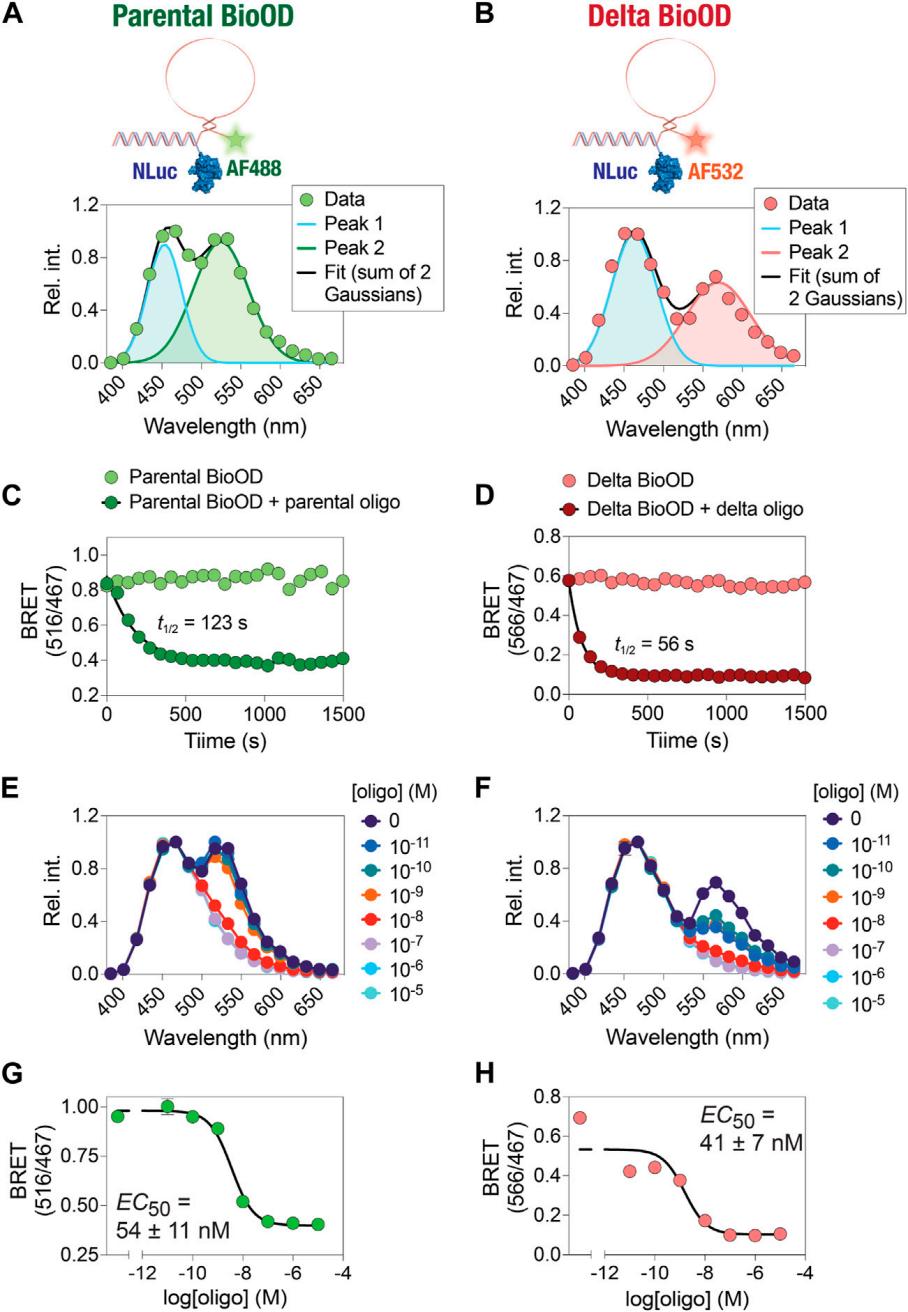
Time- and concentration-dependent change in the kinetics and affinity of the BioOD. **(A and B)** Schematic showing BRET-based parental and Delta variant BioODs. Data were fit to two Gaussian model reflecting Alexa 488 fluorescence and NLuc bioluminescence peaks **(A)** and Alexa 532 and NLuc bioluminescence peaks **(B)**. **(C and D)** Graphs showing time-dependent decrease in BRET. **(E and F)** Graphs showing the oligonucleotide concentration-dependent change in bioluminescence spectra. **(G and H)** Graphs showing the BRET of parental **(G)** and Delta variants **(H)**. Data are shown as the mean ± SD obtained from three independent experiments, with each experiment performed in triplicate.

In the second step of BioOD assembly, we incubated the constant DNA handle-conjugated NLuc(C166S/G182C) (2 µM) with 4 µM of the SARS-CoV-2 parental and Delta variant-specific molecular beacons, which contain a region complementary to the constant DNA handle at 37°C for 1 h. Following the assembly of the BioODs, we measured their bioluminescence spectra (Figures 4A and B), which revealed two emission peaks, one corresponding to NLuc (467 nm) and the other for the fluorophore, AF488 (516 nm; parental BioOD) or AF532 (566 nm; delta BioOD). The presence of the second emission peaks corresponding to the respective fluorophores indicates efficient BRET between NLuc and the fluorophores and, thus, a close spatial positioning of the two through the formation of the desired stem–loop structure of the molecular beacons.

### BioOD enables fast and high-affinity DNA oligonucleotide detection

After the assembly of the parental and delta variant BioODs, we validated them for their specificity using their specific complementary oligonucleotides (Pardee et al., 2016; Moore et al., 2021). For this, we designed two complementary DNA oligonucleotide that could bind to the entire stem–loop structure of the molecular beacons (complete complementary oligonucleotide) in the parental and Delta variant BioODs, respectively. The parental and Delta variant BioODs were incubated with their respective complete complementary oligonucleotides at a concentration of 0.2 μM at 37°C for 15 min, and the BRET (ratio of emissions at 516 nm and 467 nm for the parental BioOD since it contained AF488 as the BRET acceptor and the ratio of emissions at 566 nm and 467 nm for the Delta variant BioOD since it contained AF532 as the BRET acceptor) was measured after the addition of the luciferase substrate. Incubation of the parental and Delta variant BioODs at 37°C did not result in any discernable changes in the BRET (Figures 4C and D), suggesting that the BioODs maintain the stable stem–loop structure of the molecular beacons. In the presence of the specific, complete complementary oligonucleotides, however, both the parental and the Delta variant BioODs showed a rapid decrease in BRET (Figures 4C and D), with *t*_1/2_ of 123 s for the parental and 56 s for the Delta variant BioOD, indicating the suitability of the BioODs for the rapid detection of viral oligonucleotides.

Upon observing the fast kinetics of the BioOD response to the sequence-specific complete complementary DNA oligonucleotide, we determined the concentration-dependent response of the BioODs by incubating the parental and delta variant-specific BioODs with a range of concentrations of their respective complete complementary oligonucleotides and monitoring the bioluminescence spectra and the BRET ratio. This revealed a concentration-dependent decrease in the amplitude of the second peak, corresponding to the BRET acceptors, in the bioluminescence spectra of the BioODs (Figure 4E,F). Furthermore, fitting the BRET ratios of the BioODs obtained upon incubation with a range of complementary oligonucleotides to a sigmoidal dose–response curve revealed EC_50_ values of 54 ± 11 nM (2.5 ± 0.7 × 10^10^ copies/mL) and 41 ± 7 nM (2.5 ± 0.4 × 10^10^ copies/mL) for the parental and the Delta variant-specific BioODs, respectively (Figures 4G,H). Together, these results reveal the fast kinetics and high-affinity response of the parental and Delta variant-specific BioODs designed here. While these results show the high sensitivity of the BioODs, amplification of the viral RNA, such as through the inclusion of a step of nucleic acid sequence-based amplification (NASBA) (Berard et al., 2004), can further increase the sensitivity (Abdolahzadeh et al., 2019).

**FIGURE 5:**
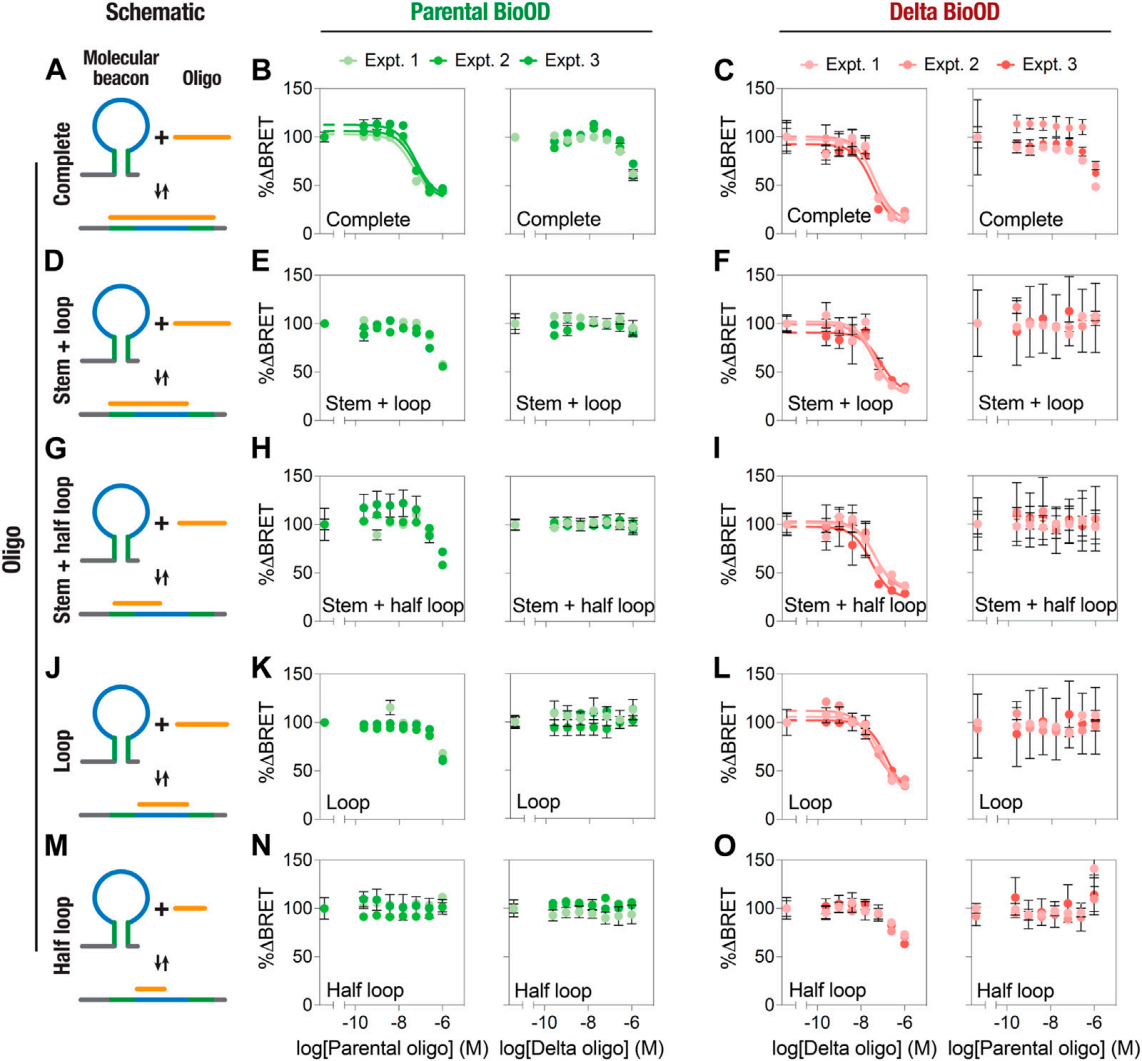
Specificity of the BRET response of parental and Delta variant-specific BioODs. **(A–O)** Schematic diagram showing the binding and the region of complementarity of complete **(A)**, stem + loop **(D)**, stem + half-loop **(G)**, loop **(J)**, and half-loop **(M)** Graphs showing the % change in BRET of the parental **(B,E,H,K,N)** and Delta variant **(C,F,I,L,O)** BioODs in the presence of the indicated concentrations of the complete **(B and C)**, stem + loop **(E and F)**, stem + half-loop **(H and I)**, loop **(K,L),** and half-loop **(N,O)** specific to the parental (**B,E,H,K,N**; left panels) and Delta variant (**B,E,H,K,N**; right panels) BioODs and Delta variant (**C,F,I,L,O**; left panel) and parental (**C,F,I,L,O**; right panel) BioODs. Data are shown as the mean ± SD from three independent experiments, with each experiment performed in triplicate.

We then performed an extensive characterization and validation of both the parental and Delta variant-specific BioODs with respect to their binding specificities and the extent of complementarity of the target oligonucleotides for their BRET response (Figure 5; Table 1). For this, we designed a number of complementary oligonucleotides specific to the parental (Supplementary Table S1) and Delta variant (Supplementary Table S2)-specific BioODs; i.e., (i) those complementary to the entire stem–loop region (including both stems) of the molecular beacons (Figure 5A), (ii) those that are complementary to the loop and one of the stem regions (Figure 5D), (iii) those that are complementary to the loop and half of the loop (Figure 5G), (iv) those that are complementary to the loop regions only (Figure 5J), and (v) those that are complementary to only half of the loop regions (Figure 5M). Each of these complementary oligonucleotides were incubated with both the parental and the Delta variant-specific BioODs at a range of concentrations, and the decrease in the BRET ratio was monitored. As reported in the previous section, both the parental and the Delta variant-specific BioODs showed a high-affinity interaction with their respective, completely complementary DNA oligonucleotides, leading to a large decrease in BRET in each case (Figure 5B,C; left panels; Table 1). On the other hand, the parental BioOD did not show such a response with the completely complementary oligonucleotide specific to the Delta variant BioOD and *vice versa*, with only some decrease in BRET observed at the highest oligonucleotide concentration of 1 μM (Figure 5B,C; right panels; Supplementary Table S3), indicating that both the parental and the Delta variant-specific BioODs are highly specific to their complementary oligonucleotides.

**TABLE 1:** Parental and Delta variant complementary oligonucleotides EC_50_ and maximum percentage BRET change.

Complementary oligonucleotides	Parental BioOD		Delta BioOD	
	EC_50_ (mean ± SD) (nM)	Maximum ΔBRET (mean ± SD) (%)	EC_50_ (mean ± SD) (nM)	Maximum ΔBRET (mean ± SD) (%)
Complete	54 ± 11	61 ± 5	40 ± 10	90 ± 10
Stem + loop	ND	44 ± 2	53 ± 20	86 ± 5
Stem + half-loop	ND	38 ± 6	37 ± 7	86 ± 4
Loop	ND	37 ± 3	81 ± 41	76 ± 9
Half-loop	ND	0	ND	30 ± 4

Having established the specific and high-affinity interaction of the BioODs against their respective complete oligonucleotides, we determined the dose–response curves for the stem–loop (Figure 5D,E,F), stem + half-loop (Figure 5G,H,I), loop (Figure 5J,K,L), and half-loop oligonucleotides (Figure 5M,N,O). The stem + loop, the stem + half-loop, and the loop oligonucleotides specific to the parental BioOD did cause a reduction in the BRET of the parental BioOD at higher concentrations, but the BRET response did not lead to saturation (Figure 5E,H,K; left panels). However, no discernable decrease in BRET of the parental BioOD was observed in the presence of the Delta variant-specific oligonucleotides (Figure 5E,H,K; right panels), suggesting that the parental BioOD does not interact with these oligonucleotides with a high affinity that could lead to a disruption of the stem–loop structure of the molecular beacon. In contrast, all these three oligonucleotides specific to the Delta variant BioOD caused a concentration-dependent decrease in the BRET of the Delta variant BioOD, with the BRET response reaching to saturation (Figure 5F,I,L; left panels), suggesting that the delta variant BioOD interacts with these oligonucleotides with relatively high affinity, leading to a loss of the stem–loop structure of the molecular beacon. However, they failed to cause any decrease in the BRET of the Delta variant BioOD (Figure 5F,I,L; right panels). Lastly, the half-loop oligonucleotide specific to the parental BioOD failed to cause any discernable change in the BRET of the BioOD (Figure 5N; left panel), while that specific to the Delta variant BioOD did show some decrease in the BRET at higher concentrations (Figure 5O; left panel), suggesting an inability of the half-loop oligonucleotide to cause a loss of the stem–loop structure of the molecular beacons in both the parental and the Delta variant BioODs.

### BioOD enables the cell phone camera-based detection of the viral DNA oligonucleotide

**FIGURE 6:**
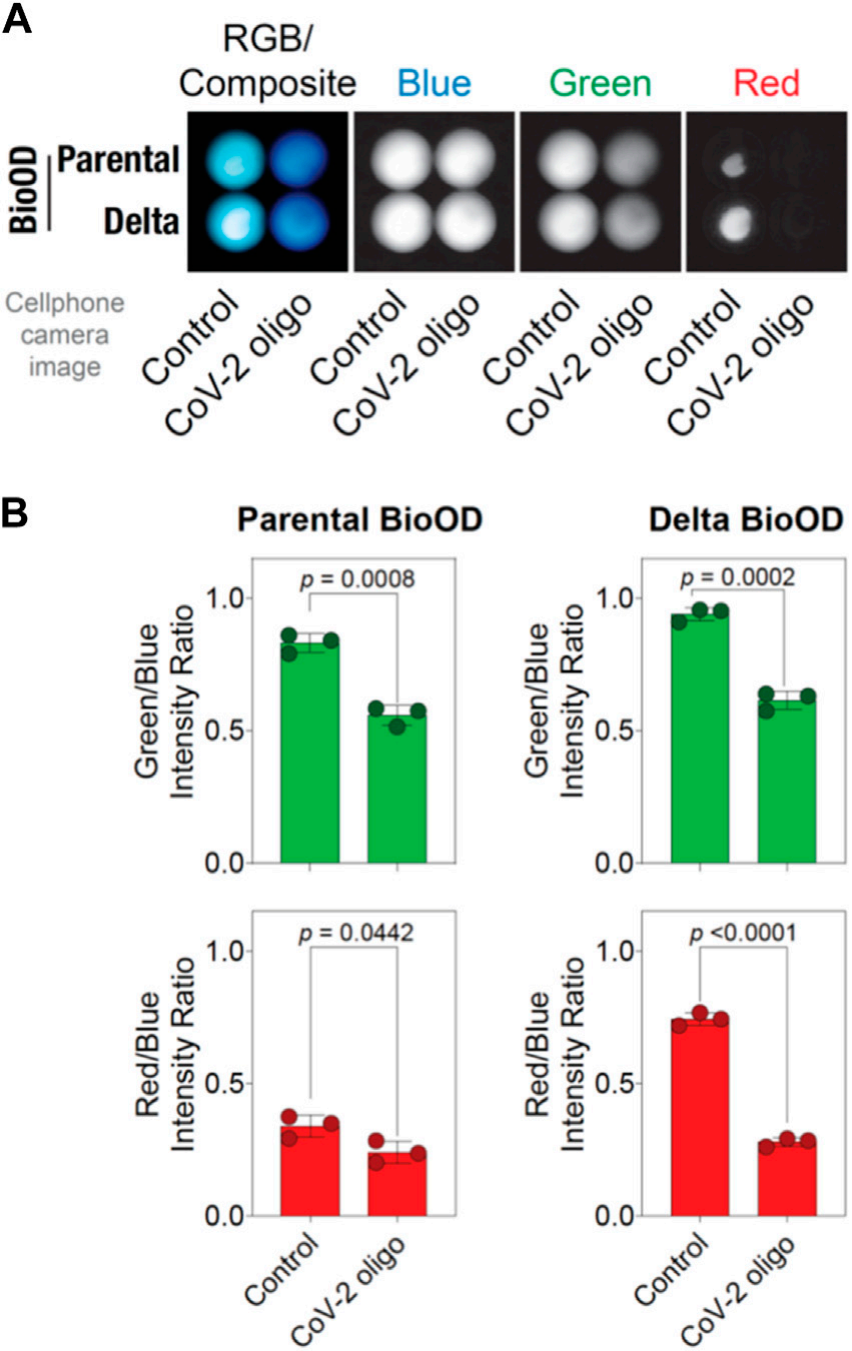
BioOD enables cell phone camera-based detection of the viral DNA oligonucleotide. **(A)** Image showing the presence and absence (control) of the complementary oligonucleotides of parental and Delta variant in the RGB composite. **(B)** Graphs showing the parental BioOD (right panel) and Delta BioOD expressing higher BRET, with green in the channel with the control, change in the BRET with blue, and less green in the presence of the complementary oligonucleotides. Graph showing the intensity ratio of red to blue in the presence of control and no change in color in the presence of complementary oligonucleotides. Data shown are the mean ± SD obtained from three independent experiments.

To extend the utility of the parental and Delta variant BioODs in a POCT setup, we attempted to determine if the BioOD showed a change in the bioluminescence spectra upon binding to the oligonucleotides complementary to their respective molecular beacons. For this, we incubated the BioODs with their respective oligonucleotides complementary to both stems and the loop of the molecular beacons used to assemble the BioODs, and bioluminescence images were acquired post-incubation using a cell phone camera upon the addition of the luciferase substrate (Figure 6A) (Hattori et al., 2020; Salimiyan rizi, 2022). As shown in Figure 6A, incubation of the BioODs resulted in a clearly observable change in the bioluminescence spectra from light blue to deep blue. Separation of the RGB image into blue, green, and red channels revealed a discernable decrease in the light intensity in the green and red channels for the BioODs (Figure 6A). This was further confirmed through quantification of the green-to-blue and red-to-blue channel intensities, which revealed a significant decrease in these ratios of both the parental and the Delta variant BioODs (Figure 6B). The BioODs developed here could be developed further for the potential utility in a POCT setup in the future.

## Conclusion

To conclude, we developed BRET-based biosensors, BioODs, as a proof-of-principle study that could be utilized for the detection of SARS-CoV-2 parental and Delta variant nucleic acid in the future. We achieved this through the combination of highly specific molecular beacons and the bright and small NLuc luciferase. Specifically, the use of the NLuc luciferase enabled the detection of the change in the bioluminescence spectra of the BioODs in the presence of their cognate DNA oligonucleotides using a regular cell phone camera, thus highlighting the possibility of using the BioODs in a POCT setup in future. We believe that the BioODs developed here may be useful in detecting SARS-CoV-2 and its Delta variant infections. While we focused on detecting SARS-CoV-2 oligonucleotides, the BioOD platform developed here can be adapted for the detection of nucleic acid, either RNA or DNA, of other viruses and pathogens, in general, through a change in the molecular beacon sequence specific to the pathogen of choice.

## Data Availability

The original contributions presented in the study are included in the article/Supplementary Material; further inquiries can be directed to the corresponding author.

## References

[B1] Abdolahzadeh A. Dolgosheina E. V. Unrau P. J. (2019). RNA detection with high specificity and sensitivity using nested fluorogenic Mango NASBA. Rna 25 (12), 1806–1813. 10.1261/rna.072629.119 31551299 PMC6859864

[B2] Ahmed W. S. Philip A. M. Biswas K. H. (2022). Decreased interfacial dynamics caused by the N501Y mutation in the SARS-CoV-2 S1 spike: ACE2 complex. Front. Mol. Biosci. 9, 846996. 10.3389/fmolb.2022.846996 35936792 PMC9355283

[B3] Altamash T. Ahmed W. Rasool S. Biswas K. H. (2021). Intracellular ionic strength sensing using NanoLuc. Int. J. Mol. Sci. 22 (2), 677. 10.3390/ijms22020677 33445497 PMC7826950

[B4] Azad T. Janse van Rensburg H. J. Morgan J. Rezaei R. Crupi M. J. F. Chen R. (2021). Luciferase-based biosensors in the era of the COVID-19 pandemic. ACS Nanosci. Au 1 (1), 15–37. 10.1021/acsnanoscienceau.1c00009 37579261 PMC8370122

[B5] Berard C. Cazalis M. A. Leissner P. Mougin B. (2004). DNA nucleic acid sequence-based amplification-based genotyping for polymorphism analysis *.* Biotechniques 37 (4), 680, 686–684, 686. 10.2144/04374dd04 15517981

[B6] Besson B. Eun H. Kim S. Windisch M. P. Bourhy H. Grailhe R. (2022). Optimization of BRET saturation assays for robust and sensitive cytosolic protein-protein interaction studies. Sci. Rep. 12 (1), 9987. 10.1038/s41598-022-12851-9 35705637 PMC9200754

[B7] Biswas K. H. Badireddy S. Rajendran A. Anand G. S. Visweswariah S. S. (2015b). Cyclic nucleotide binding and structural changes in the isolated GAF domain of Anabaena adenylyl cyclase, CyaB2. PeerJ 3, e882. 10.7717/peerj.882 25922789 PMC4411481

[B8] Biswas K. H. Groves J. T. (2016). A microbead supported membrane-based fluorescence imaging assay reveals intermembrane receptor-ligand complex dimension with nanometer precision. Langmuir 32 (26), 6775–6780. 10.1021/acs.langmuir.6b01377 27264296

[B9] Biswas K. H. Hartman K. Zaidel-Bar R. Groves J. (2016). Sustained α -catenin activation at E-cadherin junctions in the absence of mechanical force. Biophys. J. 111 (5), 1044–1052. 10.1016/j.bpj.2016.06.027 27602732 PMC5018127

[B10] Biswas K. H. Hartman K. L. Yu C. h. Harrison O. J. Song H. Smith A. W. (2015a). E-cadherin junction formation involves an active kinetic nucleation process. Proc. Natl. Acad. Sci. U. S. A. 112 (35), 10932–10937. 10.1073/pnas.1513775112 26290581 PMC4568248

[B11] Biswas K. H. Sopory S. Visweswariah S. S. (2008). The GAF domain of the cGMP-binding, cGMP-specific phosphodiesterase (PDE5) is a sensor and a sink for cGMP. Biochemistry 47 (11), 3534–3543. 10.1021/bi702025w 18293931

[B12] Biswas K. H. Visweswariah S. S. (2011). Distinct allostery induced in the cyclic GMP-binding, cyclic GMP-specific phosphodiesterase (PDE5) by cyclic GMP, sildenafil, and metal ions. J. Biol. Chem. 286 (10), 8545–8554. 10.1074/jbc.m110.193185 21193396 PMC3048737

[B13] Biswas K. H. Visweswariah S. S. (2017). Buffer NaCl concentration regulates Renilla luciferase activity and ligand-induced conformational changes in the BRET-based PDE5 sensor. Matters. 10.19185/matters.201702000015

[B14] Biswas K. H. Zhongwen C. Dubey A. K. Oh D. Groves J. T. (2018). Multicomponent supported membrane microarray for monitoring spatially resolved cellular signaling reactions. Adv. Biosyst. 2 (4), 1800015. 10.1002/adbi.201800015

[B15] Chakravarthy A. Anirudh K. N. George G. (2021). Ultrasensitive RNA biosensors for SARS-CoV-2 detection in a simple color and luminescence assay. medRxiv. 2021.01.08.21249426. 10.26508/lsa.202101213 PMC8500229 34593555

[B16] Chen M. Z. Moily N. S. Bridgford J. L. Wood R. J. Radwan M. Smith T. A. (2017). A thiol probe for measuring unfolded protein load and proteostasis in cells. Nat. Commun. 8 (1), 474. 10.1038/s41467-017-00203-5 28883394 PMC5589734

[B17] Dale N. C. Johnstone E. K. M. White C. W. Pfleger K. D. G. (2019). NanoBRET: the bright future of proximity-based assays. Front. Bioeng. Biotechnol. 7, 56. 10.3389/fbioe.2019.00056 30972335 PMC6443706

[B18] Dao Thi V. L. Herbst K. Boerner K. Meurer M. Kremer L. P. Kirrmaier D. (2020). A colorimetric RT-LAMP assay and LAMP-sequencing for detecting SARS-CoV-2 RNA in clinical samples. Sci. Transl. Med. 12 (556), eabc7075. 10.1126/scitranslmed.abc7075 32719001 PMC7574920

[B19] Dhawan M. Sharma A. Priyanka Thakur N. Rajkhowa T. K. Choudhary O. P. (2022). Delta variant (B.1.617.2) of SARS-CoV-2: mutations, impact, challenges and possible solutions. Hum. Vaccin Immunother. 18 (5), 2068883. 10.1080/21645515.2022.2068883 35507895 PMC9359381

[B20] Dong H. Mo J. Yu Y. Xie W. Zheng J. Jia C. (2023). A portable system for economical nucleic acid amplification testing. Front. Bioeng. Biotechnol. 11, 1214624. 10.3389/fbioe.2023.1214624 37600301 PMC10436208

[B21] Engelen W. van de Wiel K. M. Meijer L. H. H. Saha B. Merkx M. (2017). Nucleic acid detection using BRET-beacons based on bioluminescent protein-DNA hybrids. Chem. Commun. (Camb) 53 (19), 2862–2865. 10.1039/c6cc10032e 28217801 PMC5436041

[B22] England C. G. Ehlerding E. B. Cai W. (2016). NanoLuc: a small luciferase is brightening up the field of bioluminescence. Bioconjugate Chem. 27 (5), 1175–1187. 10.1021/acs.bioconjchem.6b00112 PMC4871753 27045664

[B23] Fang Y. Wang Y. Su X. Liu H. Chen H. Chen Z. (2022). A miniaturized and integrated dual-channel fluorescence module for multiplex real-time PCR in the portable nucleic acid detection system. Front. Bioeng. Biotechnol. 10, 996456. 10.3389/fbioe.2022.996456 36172017 PMC9510591

[B24] Feng W. Newbigging A. M. Pang B. Peng H. Cao Y. (2020). Molecular diagnosis of COVID-19: challenges and research needs. Anal. Chem. 92 (15), 10196–10209. 10.1021/acs.analchem.0c02060 32573207

[B25] Fox T. Geppert J. Dinnes J. Scandrett K. Bigio J. Sulis G. (2022). Antibody tests for identification of current and past infection with SARS-CoV-2. Cochrane Database Syst. Rev. 11 (11), Cd013652. 10.1002/14651858.cd013652.pub2 36394900 PMC9671206

[B26] Geethakumari A. M. Ahmed W. S. Rasool S. Fatima A. Nasir Uddin S. M. Aouida M. (2022a). A Genetically encoded BRET-based SARS-CoV-2 Mpro protease activity sensor. bioRxiv 5, 117. 2022.01.31.478460. 10.1038/s42004-022-00731-2 PMC9516532 36187754

[B27] Geethakumari A. M. Ahmed W. S. Rasool S. Fatima A. Nasir Uddin S. M. Aouida M. (2022b). A genetically encoded BRET-based SARS-CoV-2 M(pro) protease activity sensor. Commun. Chem. 5 (1), 117. 10.1038/s42004-022-00731-2 36187754 PMC9516532

[B28] Gil Alvaradejo G. Glassner M. Hoogenboom R. Delaittre G. (2018). Maleimide end-functionalized poly(2-oxazoline)s by the functional initiator route: synthesis and (bio)conjugation. RSC Adv. 8 (17), 9471–9479. 10.1039/c8ra00948a 35541867 PMC9078655

[B29] Gobbo P. Workentin M. S. (2012). Improved methodology for the preparation of water-soluble maleimide-functionalized small gold nanoparticles. Langmuir 28 (33), 12357–12363. 10.1021/la302168g 22881999

[B30] Grandien M. (1996). Viral diagnosis by antigen detection techniques. Clin. Diagn Virol. 5 (2-3), 81–90. 10.1016/0928-0197(96)00209-7 15566866

[B31] Guglielmi G. (2021). Rapid coronavirus tests: a guide for the perplexed. Nature 590 (7845), 202–205. 10.1038/d41586-021-00332-4 33564189

[B32] Guo C. Xu K. Chen C. Wang J. Li H. (2022). Site-specific synthesis of protein-oligo conjugates through histidine-maleimide-mediated imidazolidinone formation. Bioconjugate Chem. 33 (10), 1885–1891. 10.1021/acs.bioconjchem.2c00350 36101944

[B33] Habli Z. Saleh S. Zaraket H. Khraiche M. L. (2020). COVID-19 *in-vitro* diagnostics: state-of-the-art and challenges for rapid, scalable, and high-accuracy screening. Front. Bioeng. Biotechnol. 8, 605702. 10.3389/fbioe.2020.605702 33634079 PMC7902018

[B34] Han S.-X. Jia X. Ma J. l. Zhu Q. (2013). Molecular beacons: a novel optical diagnostic tool. Archivum Immunol. Ther. Exp. 61 (2), 139–148. 10.1007/s00005-012-0209-7 PMC7079750 23292078

[B35] Hattori M. Shirane S. Matsuda T. Nagayama K. Nagai T. (2020). Smartphone-based portable bioluminescence imaging system enabling observation at various scales from whole mouse body to organelle. Sensors 20 (24), 7166. 10.3390/s20247166 33327525 PMC7764933

[B36] He J. Zhu S. Zhou J. Jiang W. Yin L. Su L. (2022). Rapid detection of SARS-CoV-2: the gradual boom of lateral flow immunoassay. Front. Bioeng. Biotechnol. 10, 1090281. 10.3389/fbioe.2022.1090281 36704307 PMC9871317

[B37] Huang K. Martí A. A. (2012). Recent trends in molecular beacon design and applications. Anal. Bioanal. Chem. 402 (10), 3091–3102. 10.1007/s00216-011-5570-6 22159461

[B38] Iversen P. L. (2018). The threat from viruses. Mol. Basis Resil. 30, 45–76. 10.1007/978-3-319-98164-2_3

[B39] Jan Z. Geethakumari A. M. Biswas K. H. Jithesh P. V. (2023). Protegrin-2, a potential inhibitor for targeting SARS-CoV-2 main protease M(pro). Comput. Struct. Biotechnol. J. 21, 3665–3671. 10.1016/j.csbj.2023.07.020 37576748 PMC10412832

[B40] Joung J. Ladha A. Saito M. Kim N. G. Woolley A. E. Segel M. (2020). Detection of SARS-CoV-2 with SHERLOCK one-pot testing. N. Engl. J. Med. 383 (15), 1492–1494. 10.1056/nejmc2026172 32937062 PMC7510942

[B41] Ju J. Zhang X. Li L. Regmi S. Yang G. Tang S. (2022). Development of fluorescent lateral flow immunoassay for SARS-CoV-2-specific IgM and IgG based on aggregation-induced emission carbon dots. Front. Bioeng. Biotechnol. 10, 1042926. 10.3389/fbioe.2022.1042926 36312540 PMC9608551

[B42] Kabir H. B. Nam Joon C. Jay T. G. (2018). Fabrication of multicomponent, spatially segregated DNA and protein-functionalized supported membrane microarray. Langmuir 34, 9781–9788. 10.1021/acs.langmuir.8b01364 30032610

[B43] Kibbe W. A. (2007). OligoCalc: an online oligonucleotide properties calculator. Nucleic Acids Res. 35 (Web Server issue), W43–W46. 10.1093/nar/gkm234 17452344 PMC1933198

[B44] Kim Y. Sohn D. Tan W. (2008). Molecular beacons in biomedical detection and clinical diagnosis. Int. J. Clin. Exp. Pathol. 1 (2), 105–116. 18784800 PMC2480550

[B45] Li Q. Wu J. Nie J. Zhang L. Hao H. Liu S. (2020). The impact of mutations in SARS-CoV-2 spike on viral infectivity and antigenicity. Cell 182 (5), 1284–1294 e9. 10.1016/j.cell.2020.07.012 32730807 PMC7366990

[B46] Mao R. Wu X. Miao Q. Cai T. (2022). Asymmetric stem-loop-mediated isothermal amplification of nucleic acids for DNA diagnostic assays by simple modification of canonical PCR primers. Front. Bioeng. Biotechnol. 10, 931770. 10.3389/fbioe.2022.931770 35935482 PMC9355699

[B47] Marchán V. (2006). Diels-Alder cycloadditions in water for the straightforward preparation of peptide–oligonucleotide conjugates. Nucleic Acids Res. 34 (3), e24. 10.1093/nar/gnj020 16478710 PMC1369286

[B48] Mattioli C. Gourdon A. Group N. (2015). Maleimides designed for self-assembly and reactivity on graphene. Molecules 20 (10), 18856–18869. 10.3390/molecules201018856 26501250 PMC6331833

[B49] Monroy-Contreras R. Vaca L. (2011). Molecular beacons: powerful tools for imaging RNA in living cells. J. Nucleic Acids 2011, 1–15. 10.4061/2011/741723 PMC3163130 21876785

[B50] Moore K. J. M. Cahill J. Aidelberg G. Aronoff R. Bektaş A. Bezdan D. (2021). Loop-mediated isothermal amplification detection of SARS-CoV-2 and myriad other applications. J. Biomol. Tech. 32 (3), 228–275. 10.7171/jbt.21-3203-017 35136384 PMC8802757

[B51] Moovarkumudalvan B. Geethakumari A. M. Ramadoss R. Biswas K. H. Mifsud B. (2022). Structure-based virtual screening and functional validation of potential hit molecules targeting the SARS-CoV-2 main protease. Biomolecules 12 (12), 1754. 10.3390/biom12121754 36551182 PMC9775371

[B52] Nelson-Mora J. Rubio D. Ventura-Martínez A. González L. A. Del-Rio D. Aranda-López Y. (2023). New detection method of SARS-CoV-2 antibodies toward a point-of-care biosensor. Front. Bioeng. Biotechnol. 11, 1202126. 10.3389/fbioe.2023.1202126 37485316 PMC10359622

[B53] Ong T. T. Ang Z. Verma R. Koean R. Tam J. K. C. Ding J. L. (2020). pHLuc, a ratiometric luminescent reporter for *in vivo* monitoring of tumor acidosis. Front. Bioeng. Biotechnol. 8, 412. 10.3389/fbioe.2020.00412 32457886 PMC7225611

[B54] Owczarzy R. Tataurov A. V. Wu Y. Manthey J. A. McQuisten K. A. Almabrazi H. G. (2008). IDT SciTools: a suite for analysis and design of nucleic acid oligomers. Nucleic Acids Res. 36 (Web Server issue), W163–W169. 10.1093/nar/gkn198 18440976 PMC2447751

[B55] Pardee K. Green A. A. Takahashi M. K. Braff D. Lambert G. Lee J. W. (2016). Rapid, low-cost detection of zika virus using programmable biomolecular components. Cell 165 (5), 1255–1266. 10.1016/j.cell.2016.04.059 27160350

[B56] Paris C. Brun O. Pedroso E. Grandas A. (2015). Exploiting protected maleimides to modify oligonucleotides, peptides and peptide nucleic acids. Molecules 20 (4), 6389–6408. 10.3390/molecules20046389 25867825 PMC6272179

[B57] Philip A. M. Ahmed W. S. Biswas K. H. (2023). Reversal of the unique Q493R mutation increases the affinity of Omicron S1-RBD for ACE2. Comput. Struct. Biotechnol. J. 21, 1966–1977. 10.1016/j.csbj.2023.02.019 36936816 PMC10006685

[B58] Qin Z. Peng R. Baravik I. K. Liu X. (2020). Fighting COVID-19: integrated micro- and nanosystems for viral infection diagnostics. Matter 3 (3), 628–651. 10.1016/j.matt.2020.06.015 32838297 PMC7346839

[B59] Salimiyan rizi K. (2022). The smartphone biosensors for point-of-care detection of human infectious diseases: overview and perspectives—a systematic review. Curr. Opin. Electrochem. 32, 100925. 10.1016/j.coelec.2021.100925

[B60] Severins I. Szczepaniak M. Joo C. (2018). Multiplex single-molecule DNA barcoding using an oligonucleotide ligation assay. bioRxiv, 265215. 10.1016/j.bpj.2018.08.013 PMC6139880 30195940

[B61] Sherrill-Mix S. Hwang Y. Roche A. M. Glascock A. Weiss S. R. Li Y. (2021). Detection of SARS-CoV-2 RNA using RT-LAMP and molecular beacons. Genome Biol. 22 (1), 169. 10.1186/s13059-021-02387-y 34082799 PMC8173101

[B62] Sun S. Yang X. Wang Y. Shen X. (2016). *In vivo* analysis of protein–protein interactions with bioluminescence resonance energy transfer (BRET): progress and prospects. Int. J. Mol. Sci. 17 (10), 1704. 10.3390/ijms17101704 27727181 PMC5085736

[B63] Tegally H. Wilkinson E. Lessells R. J. Giandhari J. Pillay S. Msomi N. (2021). Sixteen novel lineages of SARS-CoV-2 in South Africa. Nat. Med. 27, 440–446. 10.1038/s41591-021-01255-3 33531709

[B64] Thompson J. D. Higgins D. G. Gibson T. J. (1994). CLUSTAL W: improving the sensitivity of progressive multiple sequence alignment through sequence weighting, position-specific gap penalties and weight matrix choice. Nucleic Acids Res. 22 (22), 4673–4680. 10.1093/nar/22.22.4673 7984417 PMC308517

[B65] Torretta S. Zuccotti G. Cristofaro V. Ettori J. Solimeno L. Battilocchi L. (2021). Diagnosis of SARS-CoV-2 by RT-PCR using different sample sources: review of the literature. Ear Nose Throat J. 100 (2_Suppl. l), 131s–138s. 10.1177/0145561320953231 32865458 PMC7459180

[B66] Tyagi S. Kramer F. R. (1996). Molecular beacons: probes that fluoresce upon hybridization. Nat. Biotechnol. 14 (3), 303–308. 10.1038/nbt0396-303 9630890

[B67] Van Ngoc H. Quyen T. L. Vinayaka A. C. Bang D. D. Wolff A. (2022). Point-of-care system for rapid real-time detection of SARS-CoV-2 virus based on commercially available Arduino platforms. Front. Bioeng. Biotechnol. 10, 917573. 10.3389/fbioe.2022.917573 35992344 PMC9385952

[B68] Varlamov D. A. Blagodatskikh K. A. Smirnova E. V. Kramarov V. M. Ignatov K. B. (2020). Combinations of PCR and isothermal amplification techniques are suitable for fast and sensitive detection of SARS-CoV-2 viral RNA. Front. Bioeng. Biotechnol. 8, 604793. 10.3389/fbioe.2020.604793 33251206 PMC7672014

[B69] Vet J. A. Marras S. A. (2005). Design and optimization of molecular beacon real-time polymerase chain reaction assays. Methods Mol. Biol. 288, 273–290. 10.1385/1-59259-823-4:273 15333910

[B70] Voloch C. M. da Silva Francisco R. de Almeida L. G. P. Cardoso C. C. Brustolini O. J. Gerber A. L. (2021). Genomic characterization of a novel SARS-CoV-2 lineage from Rio de Janeiro, Brazil. J. Virology 95 (10), e00119–e00121. 10.1128/jvi.00119-21 33649194 PMC8139668

[B71] Wang Y. Wang X. Chen H. Han L. Wang L. Chen T. (2021). A novel real-time reverse transcription loop-mediated isothermal amplification detection platform: application to diagnosis of COVID-19. Front. Bioeng. Biotechnol. 9, 748746. 10.3389/fbioe.2021.748746 34746104 PMC8569142

[B72] Wu F. Zhao S. Yu B. Chen Y. M. Wang W. Song Z. G. (2020). A new coronavirus associated with human respiratory disease in China. Nature 579 (7798), 265–269. 10.1038/s41586-020-2008-3 32015508 PMC7094943

[B73] Younes N. Al-Sadeq D. W. Al-Jighefee H. Younes S. Al-Jamal O. Daas H. I. (2020). Challenges in laboratory diagnosis of the novel coronavirus SARS-CoV-2. Viruses 12 (6), 582. 10.3390/v12060582 32466458 PMC7354519

[B74] Zhan Y. Yin H. Yin J. Y. (2022). B.1.617.2 (Delta) Variant of SARS-CoV-2: features, transmission and potential strategies. Int. J. Biol. Sci. 18 (5), 1844–1851. 10.7150/ijbs.66881 35342345 PMC8935235

[B75] Zhu Z. Lian X. Su X. Wu W. Marraro G. A. Zeng Y. (2020). From SARS and MERS to COVID-19: a brief summary and comparison of severe acute respiratory infections caused by three highly pathogenic human coronaviruses. Respir. Res. 21 (1), 224. 10.1186/s12931-020-01479-w 32854739 PMC7450684

[B76] Zuker M. (2003). Mfold web server for nucleic acid folding and hybridization prediction. Nucleic Acids Res. 31 (13), 3406–3415. 10.1093/nar/gkg595 12824337 PMC169194

