# Supplementary material for: BRET-based biosensors for SARS-CoV-2 oligonucleotide detection
Source: Front Bioeng Biotechnol. 2024 Jun 3;12:1353479. doi: 10.3389/fbioe.2024.1353479 (PMC11181354; doi:10.3389/fbioe.2024.1353479)
Supplement: Supplementary file 1 [file DataSheet1.docx]

# **Supporting Information**

­

**BRET-based Biosensors for SARS-CoV-2**

**Oligonucleotide Detection (BioOD)**

Asfia Sultana^1^, Anupriya M Geethakumari^1^, Zeyaul Islam^2^, Prasanna R Kolatkar^2^, Kabir H Biswas^1,*^

Affiliations:

^1^Division of Biological and Biomedical Sciences, College of Health & Life Sciences, Hamad Bin Khalifa University, Education City, Qatar Foundation, Doha – 34110, Qatar

^2^Qatar Biomedical Research Institute, Hamad Bin Khalifa University, Education City, Qatar Foundation, Doha – 34110, Qatar

*Correspondence: [kbiswas@hbku.edu.qa](mailto:kbiswas@hbku.edu.qa)

## Supporting Tables

**Supporting Table S1. Parental BioOD and its complementary oligonucleotide sequences**

| **SL.NO** | **5’-3’ Parental BioOD complementary oligonucleotide sequences** |
| --- | --- |
| Complete | AAGGAACAGCCGCTATTAACTATTAACGCTGT |
| Stem + loop | AAGGAACAGCCGCTATTAACTATTAAC |
| Stem + half loop | AAGGAACAGCCGCTATTAAC |
| Loop | CGCTATTAACTATTAAC |
| Half loop | CGCTATTAACATAATT |
| PARENT-BioOD | TTCCTCTACCACCTACATCACACAGCGTTAATAGTTAATAGCGGCTGTTCCTT |

**Supporting Table S2. Delta BioOD and its complementary oligonucleotide sequences**

| **SL.NO** | **5’-3’ Delta BioOD complementary oligonucleotide sequences** |
| --- | --- |
| Complete | AAGGAACAGCTTTGAAGTTTTCCAAGTGCGCTGT |
| Stem + loop | AAGGAACAGCTTTGAAGTTTTCCAAGTGC |
| Stem + half loop | AAGGAACAGCTTTGAAGTTTTC |
| Loop | TTTGAAGTTTTCCAAGTGC |
| Half loop | TTTGAAGTTTTCGTTC (EXTRA NUCLEOTIDES) |
| DELTA-BioOD | TTCCTCTACCACCTACATCACACAGCGCACTTGGAAAACTTCAA**A**GCTGTTCCTT |

**Supporting Table S3. EC50 and maximum %BRET change of parental and delta BioOD with interchange complementary oligonucleotide (Interchange assay)**

| **Complementary oligonucleotide** | **Parental BioOD** | | **Delta BioOD** | |
| --- | --- | --- | --- | --- |
|  | **Delta oligonucleotide (Interchange assay)** | | **Parental oligonucleotide (Interchange assay)** | |
|  | **EC_50_ ± SD (nM)** | **Maximum**  **% ΔBRET**  **(mean ± SD)** | **EC_50_ ± SD (nM)** | **Maximum**  **% ΔBRET**  **(mean ± SD)** |
| Complete | ND | 64 ± 5 | 35 ± 30 | 61 ± 9 |
| Stem + loop | ND | ND | ND | ND |
| Stem + half loop | ND | ND | ND | ND |
| Loop | ND | ND | ND | ND |
| Half loop | ND | ND | ND | ND |

Supporting Text

1. **SNAP-GLuc-Xa-His10 Fusion construct:**

**
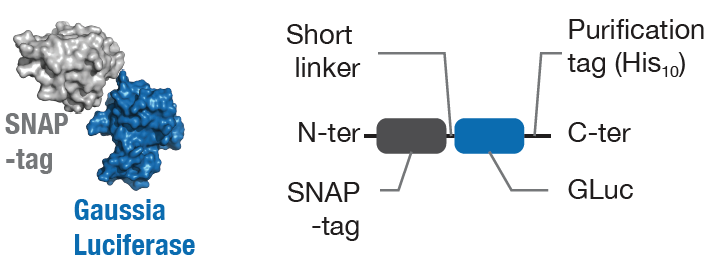
**

*Protein sequence*:

MDKDCEMKRTTLDSPLGKLELSGCEQGLHEIKLLGKGTSAADAVEVPAPAAVLGGPEPLMQATAWLNAYFHQPEAIEEFPVPALHHPVFQQESFTRQVLWKLLKVVKFGEVISYQQLAALAGNPAATAAVKTALSGNPVPILIPCHRVVSSSGAVGGYEGGLAVKEWLLAHEGHRLGKPGLGGSGSTGKPTENNEDFNIVAVASNFATTDLDADRGKLPGKKLPLEVLKEMEANARKAGCTRGCLICLSHIKCTPKMKKFIPGRCHTYEGDKESAQGGIGEAIVDIPAIPRFKDLEPMEQFIAQVDLCVDCTTGCLKGLANVQCSDLLKKWLPQRCATFASKIQGQVDKIKGAGGDIEGRHHHHHHHHHH

*Nucleotide sequence*:

ATGGACAAAGATTGCGAAATGAAACGTACCACCCTGGATAGCCCGCTGGGCAAACTGGAACTGAGCGGCTGCGAACAGGGCCTGCATGAAATTAAACTGCTGGGTAAAGGCACCAGCGCGGCCGATGCGGTTGAAGTTCCGGCCCCGGCCGCCGTGCTGGGTGGTCCGGAACCGCTGATGCAGGCGACCGCGTGGCTGAACGCGTATTTTCATCAGCCGGAAGCGATTGAAGAATTTCCGGTTCCGGCGCTGCATCATCCGGTGTTTCAGCAGGAGAGCTTTACCCGTCAGGTGCTGTGGAAACTGCTGAAAGTGGTTAAATTTGGCGAAGTGATTAGCTATCAGCAGCTGGCGGCCCTGGCGGGTAATCCGGCGGCCACCGCCGCCGTTAAAACCGCGCTGAGCGGTAACCCGGTGCCGATTCTGATTCCGTGCCATCGTGTGGTTAGCTCTAGCGGTGCGGTTGGCGGTTATGAAGGTGGTCTGGCGGTGAAAGAGTGGCTGCTGGCCCATGAAGGTCATCGTCTGGGTAAACCGGGTCTGGGAGGCTCTGGCTCTACTGGCAAACCAACTGAAAACAATGAAGATTTCAACATTGTAGCTGTAGCTAGCAACTTTGCTACAACGGATCTCGATGCTGACCGTGGTAAATTGCCCGGAAAAAAATTACCACTTGAGGTACTCAAAGAAATGGAAGCCAATGCTAGGAAAGCTGGCTGCACTAGGGGATGTCTGATATGCCTGTCACACATCAAGTGTACACCCAAAATGAAGAAGTTTATCCCAGGAAGATGCCACACCTATGAAGGAGACAAAGAAAGTGCACAGGGAGGAATAGGAGAGGCTATTGTTGACATTCCTGCAATTCCTCGGTTTAAGGATTTGGAACCGATGGAACAATTCATTGCACAAGTTGACCTATGTGTAGACTGCACAACTGGATGCCTCAAAGGTCTTGCCAATGTGCAATGTTCTGATTTACTCAAGAAATGGCTGCCACAAAGATGTGCAACTTTTGCTAGCAAAATTCAAGGCCAAGTGGACAAAATAAAGGGTGCCGGTGGTGATATCGAAGGTAGGCATCACCATCACCATCACCATCACCATCACTAA

Pink: SNAP-tag

Yellow: GS linker

Cyan: GLuc

Green: Xa cleavage site

Gray: His10

1. **NLuc(C164S/G182C)–Strep-tag-NLuc-Cys-Xa-His10**

*
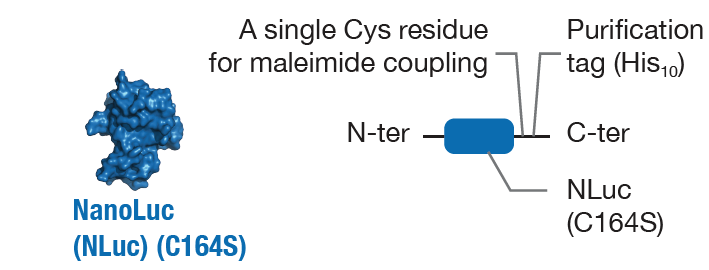
*

*Protein sequence*:

MVFTLEDFVGDWRQTAGYNLDQVLEQGGVSSLFQNLGVSVTPIQRIVLSGENGLKIDIHVIIPYEGLSGDQMGQIEKIFKVVYPVDDHHFKVILHYGTLVIDGVTPNMIDYFGRPYEGIAVFDGKKITVTGTLWNGNKIIDERLINPDGSLLFRVTINGVTGWRLSERILAAAALELPETGCGIEGRHHHHHHHHHH

*Nucleotide sequence*:

ATGGTATTTACTCTTGAAGATTTTGTCGGTGATTGGCGCCAGACCGCCGGCTATAACCTGGACCAAGTGCTTGAACAGGGCGGGGTTAGCAGCCTGTTTCAAAACCTGGGGGTGAGTGTCACGCCAATTCAGCGCATCGTTCTGTCGGGAGAGAATGGTCTGAAAATCGATATCCACGTCATTATCCCGTACGAAGGTCTTTCTGGTGATCAGATGGGGCAGATAGAAAAAATATTCAAAGTGGTGTACCCAGTAGACGATCATCACTTCAAGGTTATACTGCACTATGGCACCCTCGTTATCGATGGCGTTACTCCGAATATGATCGATTACTTTGGGCGTCCTTATGAAGGTATTGCGGTGTTCGACGGTAAAAAAATTACGGTTACCGGGACGCTCTGGAATGGTAATAAAATCATTGATGAGCGCTTGATAAACCCAGATGGCAGCCTTCTGTTCAGAGTTACGATAAACGGGGTTACGGGTTGGCGACTGAGCGAAAGAATATTAGCTGCGGCCGCACTCGAGCTGCCAGAAACCGGTTGTGGTatcgaaggtaggcatcaccatcaccatcaccatcaccatcacTAA

Cyan: NLuc(C164S/G182C)

Blue: Cys for maleimide coupling

Green: Xa cleavage site

Gray: His10

## Supporting figures


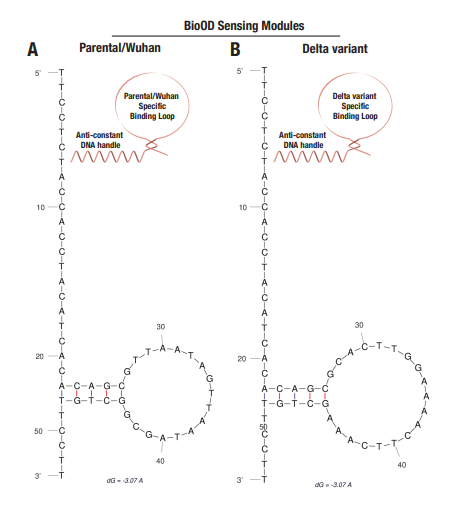


**Supporting Fig. S1.**  **A, B** Schematic showing the detailed design including DNA oligonucleotide sequence of the DNA stem-loop structures designed for the detection of the parental and the delta variant of SARS-CoV-2.


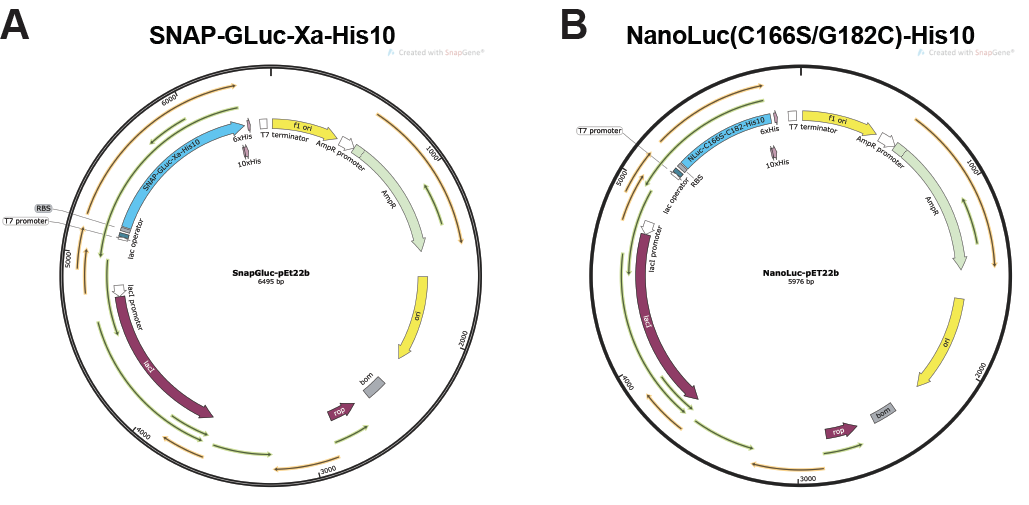


**Supporting Fig. S2. SNAP-GLuc-His10 and NLuc(C166S/G182C)-His10 proteins plasmid constructs.** (A) Plasmid constructs designed for the expression and purification of SNAP-GLuc-His10. (B) Plasmid construct of cysteine modified NLuc(C166S/G182C)-His10 protein.
